# Left ventricular hypertrophy and geometry in type 2 diabetes patients with chronic kidney disease. An echocardiographic study

**DOI:** 10.5830/CVJA-2011-028

**Published:** 2012-03

**Authors:** MP Bayauli, FB Lepira, PK Kayembe, JR M’Buyamba-Kabangu

**Affiliations:** Department of Internal Medicine, Division of Endocrinology, University of Kinshasa Hospital, Democratic Republic of the Congo (DRC); Department of Internal Medicine, Division of Nephrology, University of Kinshasa Hospital, Democratic Republic of the Congo (DRC); Department of Epidemiology and Biostatistics, Kinshasa; School of Public Health, University of Kinshasa, Democratic Republic of the Congo (DRC); Hypertension Unit, University of Kinshasa Hospital, Democratic Republic of the Congo (DRC)

**Keywords:** type 2 diabetes, chronic kidney disease, left ventricular hypertrophy, prevalence, predictors

## Abstract

**Objective:**

We assessed left ventricular structural alterations associated with chronic kidney disease (CKD) in Congolese patients with type 2 diabetes.

**Methods:**

This was a cross-sectional study of a case series. We obtained anthropometric, clinical, biological and echocardiographic measurements in 60 consecutive type 2 diabetes patients (37 females, 62%) aged 20 years or older from the diabetes outpatient clinic, University of Kinshasa Hospital, DRC. We computed creatinine clearance rate according to the MDRD equation and categorised patients into mild (CrCl > 60 ml/min per 1.73 m^2^), moderate (CrCl 30–60 ml/min per 1.73 m^2^) and severe CKD (< 30 ml/min per 1.73 m^2^). Left ventricular hypertrophy (LVH) was indicated by a LV mass index (LVMI) > 51 g/m^2.7^ and LV geometry was defined as normal, or with concentric remodelling, eccentric or concentric hypertrophy, using relative wall thickness (RWT) and LVMI.

**Results:**

Compared to patients with normal kidney function, CKD patients had higher uric acid levels (450 ± 166 vs 306 ± 107 μmol/l; *p* ≤ 0.001), a greater proportion of LVH (37 vs 14%; *p* ≤ 0.05) and longstanding diabetes (13 ± 8 vs 8 ± 6 years; *p* ≤ 0.001). Their left ventricular internal diameter, diastolic (LVIDD) was (47.00 ± 6.00 vs 43.00 ± 7.00 mm; *p* ≤ 0.001), LVMI was (47 ± 19 vs 36.00 ± 15 g/m^2.7^; *p* ≤ 0.05) and proportions of concentric (22 vs 11%; *p* ≤ 0.05) or eccentric (15 vs 3%; *p* ≤ 0.05) LVH were also greater. Severe CKD was associated with increased interventricular septum, diastolic (IVSD) (12.30 ± 3.08 vs 9.45 ± 1.94 mm; *p* ≤ 0.05), posterior wall thickness, diastolic (PWTD) (11.61 ± 2.78 vs 9.52 ± 1.77 mm; *p* ≤ 0.01), relative wall thickness (RWT) (0.52 ± 0.17 vs 0.40 ± 0.07; *p* ≤ 0.01) rate of LVH (50 vs 30%; *p* ≤ 0.05), and elevated proportions of concentric remodelling (25 vs 15%; *p* ≤ 0.05) and concentric LVH (42 vs 10%; *p* ≤ 0.05) in comparison with patients with moderate CKD. In multivariable adjusted analysis, hyperuricaemia emerged as the only predictor of the presence of LVH in patients with CKD (adjusted OR 9.10; 95% CI: 2.40–33.73).

**Conclusion:**

In keeping with a higher rate of cardiovascular events usually reported in patients with impaired renal function, CKD patients exhibited LVH and abnormal LV geometry.

## Abstract

Prevention of cardiovascular disease (CVD) requires early detection and correction of predisposing conditions and risk factors in susceptible subjects.^1^ Diabetes is a major risk factor for CVD, the prognosis of which lies not only in the level of plasma glucose but also in associated factors such as left ventricular hypertrophy (LVH).^2^ The latter develops frequently among diabetic patients, including blacks, and has been identified as a powerful marker of impaired prognosis.^2^ Besides hyperglycaemia, various conditions such as aging, hypertension, obesity, central obesity, dyslipidaemia and physical inactivity are known to alter LV structure.^2^

Several reports have indicated that chronic kidney disease (CKD) is independently associated with the presence of LVH on echocardiography, suggesting that CKD might be related to LV mass index (LVMI).^3^-^5^ Individuals with LVH have eccentric or concentric hypertrophy as a result of both pressure and volume overload.^4^ Moderate to severe CKD affects 15 to 33% of diabetic patients and predicts the occurrence of CVD.^6^,^7^ Therefore, diabetic patients with CKD might be at a high risk for LVH and subsequent CVD in comparison with those without renal dysfunction.^6^,^7^

Such an association holds more risk for black people, whose high propensity to diabetic nephropathy has often been documented.^1^ There is a need to document the impact of renal function on CV morbidity and mortality in diabetic patients with CKD, particularly blacks.^1^ The aim of the present study was to evaluate the association between CKD and LV structural alterations in a clinic-based sample of consecutive Congolese patients with type 2 diabetes mellitus.

## Methods

We enrolled in the present study consecutive type 2 diabetes subjects aged 20 years and older attending the outpatient clinic at the University of Kinshasa Hospital. Ethical approval was obtained from the institutional ethics review board and informed consent was obtained from the study participants. Exclusion criteria included ischaemic heart disease (IHD), acute coronary syndrome (ACS), congestive heart failure (CHF, NYHA class II or greater), valvular heart disease, cerebral infarction or transient ischaemic attack (TIA).

Self-reported physical activity, alcohol use and smoking habits, known duration of diabetes mellitus, current treatments and measures of adiposity [body mass index (BMI) and waist circumference] were obtained in all patients. Overweight and obesity were classified as BMI ≥ 25 kg/m^2^ and ≥ 30 kg/m^2^, respectively. Central obesity was categorised as waist circumference > 102 cm in men and > 88 cm in women.

Blood pressure (BP) was measured in the supine position using a mercury sphygmomanometer with an appropriate cuff on the left arm; the average of two readings was used for statistical analysis. Pulse pressure (PP) calculated as systolic blood pressure (SBP) minus diastolic blood pressure (DBP) was considered increased when > 60 mmHg.^8^ Hypertension was defined as BP > 140/90 mmHg or currently on antihypertensive treatment. Heart rate was counted over a full minute.

A 12-hour overnight fasting venous blood sample was collected for measurement of total cholesterol and its sub-fractions [low-density lipoprotein cholesterol (LDL-C) high-density lipoprotein cholesterol (HDL-C)], triglycerides (TG), plasma glucose, serum uric acid and creatinine levels. LDL-C was calculated according to the Friedewald formula.^9^ Dyslipidaemia was an LDL-C level ≥ 2.6 mmol/l or HDL-C < 1.03 mmol/l or TG > 1.69 mmol/l.

According to the NCEP-ATP III guidelines,^10^ the metabolic syndrome (MS) was, in addition to diabetes, the presence of two of the followings risk factors: BP > 130/85 mmHg or current antihypertensive treatment, central obesity as defined above, HDL-C < 1.03 mmol/l and/or TG > 1.69 mmol/l.

We computed glomerular filtration rate [creatinine clearance (CrCl)] using the MDRD equation.^11^ Chronic kidney disease (CKD) was a CrCl rate < 60 ml/min per 1.73 m^2^; it was stratified into mild (CrCl > 60 ml/min per 1.73 m^2^), moderate (CrCl: 30–60 ml/min per 1.73 m^2^) and severe (CrCl < 30 ml/min per 1.73 m^2^).^12^ A uric acid level > 416 μmol/l defined hyperuricaemia. Proteinuria was a 24-hour urine protein excretion rate > 0.3 g.

Echocardiographic examination was performed with the patient in the partial left lateral decubitus position using an Acuson 128XP/10˝ machine with a 3.5-MHz transducer. Two-dimensional guided M-mode measurements were obtained as recommended by the American Society of Echocardiography (ASE).^13^

We used the Devereux modified cubed formula to calculate left ventricular mass (LVM).^14^ To account for gender and body size variations, LVM was indexed to height^2^.^7^, with a boundary of 51 g/m^2^.^7^ to define LVH in both genders.^15^ Relative wall thickness (RWT) was calculated as 2 × PWTD (posterior wall thickness, diastolic)/LVIDD (left ventricular internal diameter, diastolic). It was considered increased when > 0.45.^16^ RWT and left ventricular mass index (LVMI) were used to characterise LV geometry as normal (normal LVMI and normal RWT), concentric remodelling (normal LVMI and increased RWT), concentric hypertrophy (increased LVMI and increased RWT) and eccentric hypertrophy (increased LVMI and normal RWT). LV ejection fraction (LVEF) was calculated using Tiechloz’s formula.^17^

## Statistical analysis

Data are expressed as mean ± standard deviation (SD) or relative frequency in per cent. The distribution of duration of hypertension and triglyceride levels being positively skewed, the non-parametric Mann-Whitney test was used for these variables. Chi-square and Student *t*-tests were used for comparing categorical and normally distributed continuous variables, respectively.

Multiple regression models and the likelihood ratio method were performed with LVH as the dependent variable for the assessment of the strength and independence of association with risk factors. Adjusted odds ratio (aOR) were calculated for each variable from a model which included all these variables; the resulting aOR allowed the direct comparison of the independent effects of these variables to decide which variable has the greater effect on LVH. All statistical analyses were performed with SPSS for Windows, version 18.0. A *p*-value ≤ 0.5 was considered statistically significant.

## Results

Tables 1 and 2 show clinical and biological characteristics of patients according to renal function. Mean age and duration of diabetes were 58 ± 8 and 11 ± 8 years, respectively for the whole group. BMI, waist circumference, SBP and DBP, and plasma glucose levels averaged 26 ± 5 kg/m^2^, 95 ± 12 cm, 148 ± 26 mmHg, 84 ± 13 mmHg, and 8.10 ± 3.31 mmol/l, respectively. Diabetes was frequently associated with other CV risk factors, among which hypertension (80%) was the commonest. Clustering of risk factors into the metabolic syndrome was observed in 58% of patients.

**Table 1:** Clinical Characteristics Of The Whole Group And Diabetics With And Without CKD

*Characteristic*	*Whole group (n = 60)*	*Normal renal function (n = 28)*	*CKD (n = 32)*
Gender: M/F	23/37	11/17	12/20
Age (years)	58 ± 8	59 ± 8	58 ± 8
Duration DM (years)	11 ± 8	8 ± 6	13 ± 8***
Central obesity (%)	62	32	30
AHT (%)	80	37	43
MS (%)	58	28	30
Antidiabetic drugs (%)	97	45	52
Antihypertensive drugs (%)	67	25	42**
Smoking (%)	10	7	3
BMI (kg/m^2^)	26 ± 5	27 ± 6	26 ± 5
Waist (cm)	95 ± 12	96 ± 13	95 ± 12
SBP (mmHg)	148 ± 26	148 ± 29	149 ± 23
DBP (mmHg)	84 ± 13	86 ± 15	82 ± 11
PP (mmHg)	64 ± 21	61 ± 23	67 ± 19
Heart rate (beats/min)	83 ± 15	83 ± 20	86 ± 12

Data are expressed as mean ± SD or relative frequency in per cent. CKD, chronic kidney disease; M, male; F, female; DM, diabetes mellitus; AHT, arterial hypertension; MS, metabolic syndrome; BMI, body mass index; SBP, systolic blood pressure; DBP, diastolic blood pressure; PP, pulse pressure. *p ≤ 0.05; **p ≤ 0.01; ***p ≤ 0.001in comparison with normal renal function.

**Table 2:** Biological Characteristics Of The Patients And Data According To Renal Function

*Characteristic*	*Whole group (n = 60)*	*Normal renal function (n = 28)*	*CKD (n = 32)*
TC (mmol/l)	5.61 ± 1.62	5.74 ± 1.40	5.62 ± 1.71
LDL-C (mmol/l)	3.80 ± 1.54	3.90 ± 1.42	3.73 ± 0.78
HDL-C (mmol/l)	1.45 ± 0.67	1.44 ± 0.51	1.53 ± 0.84
TG (mmol/l)	1.60 ± 1.30	1.84 ± 1.80	1.33 ± 0.78
Glucose (mmol/l)	8.10 ± 3.31	8.27 ± 2.77	7.80 ± 3.80
Uric acid (μmol/l)	410 ± 178	309 ± 107	500 ± 166***
CrCl (ml/min/173 m^2^)	64 ± 41	97 ± 35	35 ± 18
24-h proteinuria (g)	0.79 ± 1.72	0.07 ± 0.164	1,42 ± 2,17

Data are expressed as mean ± SD CKD, chronic kidney disease; TC, total cholesterol; LDL-C, low-density lipoprotein cholesterol; HDL-C, high-density lipoprotein cholesterol; TG, triglycerides; CrCl, creatinine clearance. *p ≤ 0.05; **p ≤ 0.01; ***p ≤ 0.001 in comparison with normal renal function.

Besides antidiabetic therapy, 97% of patients were receiving BP-lowering drugs. CKD was observed in 32 patients (53%), 20 of whom (62%) had a CrCl rate of 30 ml/min per 1.73 m^2^ or higher. Compared to those with normal renal function, the duration of diabetes was longer (13 ± 8 vs 8 ± 6 years; *p* ≤ 0.001), the proportion of patients on current antihypertensive drugs greater (42 vs 25%; *p* < 0.05) and the level of uric acid higher (450 ± 166 vs 306 ± 107 μmol/l; *p* ≤ 0.001) in CKD patients. The two subgroups were similar for the other variables.

Table 3 summarises echocardiographic measurements by renal function status and Table 4 by the severity of renal dysfunction. Patients with CKD had increased LVIDD (47.00 ± 6.00 vs 43.00 ± 7.00 mm; *p* ≤ 0.001), LVMI (47.00 ± 19 vs 36.00 ± 15.00 mm; *p* ≤ 0.05) and higher proportions of LVH (37 vs 14%; *p* ≤ 0.05); they also showed higher proportions of concentric (22 vs 11%; *p* ≤ 0.05) and eccentric (15 vs 3%; *p* ≤ 0.05) LVH. Compared to patients with moderate CKD, those with severe CKD had increased interventricular septum thickness, diastolic (IVSD) (12.30 ± 3.08 vs 9.45 ± 1.94 mm; *p* ≤ 0.001), RWT (0.52 ± 0.17 vs 0.40 ± 0.07 mm; *p* ≤ 0.01) and higher proportions of LVH (50 vs 30%; *p* ≤ 0.05). Concentric remodelling (25 vs 15%; *p* ≤ 0.05) and concentric hypertrophy (42 vs 10%; *p* ≤ 0.05) were the geometric patterns most frequently encountered in patients with severe CKD. Between groups, systolic function indices did not differ.

**Table 3:** M-Mode Echocardiographic Data In The Whole Group And Diabetics With And Without CKD

*Characteristic*	*Whole group (n = 60)*	*Normal renal function (n = 28)*	*CKD (n = 32)*
LV dimension			
LVIDD (mm)	44.83 ± 6.62	43.00 ± 7.00	47.0 ± 6.00***
LVIDS (mm)	29.67 ± 8.43	28.04 ± 7.72	31.09 ± 8.98
IVSD (mm)	10.42 ± 2.60	10.30 ± 2.41	11.00 ± 3.48
PWTD (mm)	9.98 ± 2.26	10.00 ± 2.00	10.00 ± 2.30
RWT	0.46 ± 0.13	0.47 ± 0.12	0.45 ± 0.13
LVMI (g/m^2.7^)	41.83 ± 17.72	36.00 ± 15.00	47.00 ± 19.00*
EF (%)	68.25 ± 19.06	69.24 ± 17.02	67.39 ± 20.91
FS (%)	0.34 ± 0.13	0.34 ± 0.13	0.34 ± 0.14
LV geometry			
Normal (%)	43	43	44
Concentric remodelling (%)	30	43	19*
Concentric hypertrophy (%)	17	11	22*
Eccentric hypertrophy (%)	10	3	15*

Data are expressed as mean ± SD or relative frequency in per cent. CKD, chronic kidney disease; LVIDD, left ventricular internal diameter, diastolic; LVIDS, left ventricular internal diameter, systolic; IVSD, interventricular septum, diastolic; PWTD, posterior wall thickness, diastolic; RWT, relative wall thickness; LVMI, left ventricular mass index; EF, ejection fraction; FS, fraction shortening. *p ≤ 0.05; **p ≤ 0.01; ***p ≤ 0.001 in comparison with normal renal function.

**Table 4:** Severity Of Renal Dysfunction And M-Mode Echocardiographic Data Among Diabetics With CKD

	*CrCl 30–60 ml/ min (n = 20)*	*CrCl < 30 ml/ min (n = 12)*
LV dimension			
LVIDD (mm)	46.75 ± 5.72	46.75 ± 6.79
LVIDS (mm)	30.50 ± 9.76	32.08 ± 7.80
IVSD (mm)	9.45 ± 1.94	12.30 ± 3.08*
PWTD (mm)	9.52 ± 1.77	11.61 ± 2.78**
RWT	0.40 ± 0.07	0.52 ± 0.17**
LVMI (g/m^2.7^)	43.52 ± 15.74	52.41 ± 22.40
FS (%)	0.35 ± 0.15	0.32 ± 0.11
LV geometry			
Normal, %	55	25*
Concentric remodelling (%)	15	25*
Concentric hypertrophy (%)	10	42*
Eccentric hypertrophy (%)	20	8*

Data are expressed as mean ± SD or relative frequency in per cent. CKD, chronic kidney disease; CrCl, creatinine clearance; LVIDD, left ventricular internal diameter, diastolic; LVIDS, left ventricular internal diameter, systolic; IVSD, interventricular septum, diastolic; PWTD, posterior wall thickness, diastolic; RWT, relative wall thickness; LVMI, left ventricular mass index; EF, ejection fraction; FS, fraction shortening. *p ≤ 0.05; **p ≤ 0.01; ***p ≤ 0.001 in comparison with moderate CKD.

In multivariable adjusted analysis, the probability of LVH among CKD patients was increased by hyperuricaemia (aOR 9.10; 95% CI: 2.40–33.74) for the presence versus the absence of hyperuricaemia.

## Discussion

The key finding of the study was that the elevated prevalence of chronic kidney disease in our diabetic patients was associated with abnormal cardiac structure. The alteration in renal function was moderate in the majority of cases. Left ventricular mass index, the frequency of left ventricular hypertrophy and uric acid levels were higher in CKD patients in whom multivariable adjusted analysis indicated uric acid as the only predictor of LVH.

The elevated prevalence of moderate to severe CKD has been reported in 15 to 23% of diabetic patients in whom it predicts the occurrence of CVD.^18^,^19^ The mechanisms by which chronic hyperglycaemia may induce cardiovascular and renal dysfunction include enhanced polyol pathway flux, altered redox state, increased formation of diacylglycerol (DAG) and subsequent activation of protein kinase C (PKC) isoforms, and accelerated non-enzymatic formation of advanced glycation end products (AGEs).^20^ The DAG–PKC pathway affects cardiovascular and renal structure and function in many ways, e.g. the regulation of endothelial permeability, vascular tone, cell growth, angiogenesis, and cytokine and leucocyte activation.^20^ Moreover, insulin resistance/hyperinsulinaemia-induced activation of the sympathetic nervous and renin–angiotensin–aldosterone systems could contribute to cardiovascular and renal damage through oxidative stress and inflammation.^21^-^23^

Alteration in kidney function predominated in patients with a longer duration of diabetes, enhancing the effect of both chronic hyperglycaemia and the ageing process.^3^,^24^ The latter is associated with changes in vascular structure and function due to clustering of multiple risk factors, including insulin resistance/hyperinsulinaemia, oxidative stress and inflammation.^25^ Arterial stiffness, an independent predictor of morbidity and mortality, has been reported to increase with age and is associated with high systolic and pulse pressure.^3^,^24^,^26^ Moreover, the decrease in the number of nephrons, which occurs with ageing, may result in hyperfiltration, hypertrophy and elevation in glomerular capillary pressure.^27^

The high prevalence of LVH in CKD patients found in our study agrees with that of other studies.^28^-^31^ LVH in CKD is thought to result partly from uraemia-associated risk factors such as anaemia, calcium-phosphate products and hyperhomocysteinaemia.^4^ Moreover, renal dysfunction activates the renin–angiotensine–aldosterone system, with subsequent formation of angiotensin II, known to be essential for the development and progression of LVH.^32^ The risk of CVD and death increases with the decline in glomerular filtration rate (GFR) and the major increase in risk occurs at a GFR < 60 ml/min per 1.73 m^2^.^4^

LVH has been reported to predispose to ischaemic heart disease, arrhythmias and congestive heart failure.^33^ Our results indicate that patients with severe CKD had higher proportions of abnormal LV geometry, with concentric remodelling and concentric hypertrophy as the most frequent pattern. Both eccentric and concentric hypertrophy may occur in individuals with CKD.^4^ Eccentric hypertrophy is thought to result from volume overload, leading to cardiomyocyte drop out. Concentric hypertrophy is typically the result of hypertension and increased afterload and is exacerbated by anaemia, hyperparathyroidism and high angiotensin II concentrations. Eccentric and concentric hypertrophy have different impacts on the prognosis.^4^ Concentric hypertrophy confers the worst prognosis, followed by eccentric hypertrophy and concentric remodelling.^33^

Moderate CKD and a high proportion of hypertension could explain the pre-eminence of concentric hypertrophy observed in the present study. In Nigerian hypertensive patients, Aje *et al.* reported greater systolic, diastolic, pulse and mean blood pressure among patients with concentric hypertrophy in comparison with those with normal geometric patterns.^34^

In our study, hyperuricaemia emerged as the only predictor of LVH in CKD patients. The mechanisms that could account for increased uric acid levels in CKD include overproduction to counteract oxidative stress and endothelial dysfunction, the severity of diabetes and/or hypertension, impaired renal uric acid clearance, and insulin resistance/hyperinsulinaemia-induced proximal renal tubular reabsorption of sodium and urate.^35^,^36^ The association between hyperuricaemia and LVH could rely upon an association of uric acid with other risk factors, either isolated or combined in the metabolic syndrome.^32^ The coexistence of hyperuricaemia and LVH has been recognised as an independent and powerful predictor of CVD.^36^-^38^

The interpretation of the results of our study is confounded by some limitations. The cross-sectional design of the work precludes any causal relationship between CKD and associated risk factors. Moreover, the sample size did not allow sufficient power to detect additional associations. One wonders to what extent the conclusions of this clinic-based study could be extrapolated to the general population, given the bias in the referral of patients. The findings of our study bear, however, some clinical implications for CKD identification, treatment protocol and estimated prognosis in hypertensive patients.

## Conclusion

This study has shown that LVH is common among type 2 diabetes patients with CKD. Concentric LVH was the geometric LV pattern most frequently encountered and its frequency increased with the decline in renal function. Hyperuricaemia emerged as the unique independent predictor of the risk of LVH
